# Prospective Associations of Hemoglobin A_1c_ and c-peptide with Risk of Diabetes-related Cancers in the Cancer Prevention Study-II Nutrition Cohort

**DOI:** 10.1158/2767-9764.CRC-22-0082

**Published:** 2022-07-14

**Authors:** Peter T. Campbell, Christina C. Newton, Eric J. Jacobs, Marjorie L. McCullough, Ying Wang, Erika Rees-Punia, Mark A. Guinter, Neil Murphy, Jill Koshiol, Ahmed N. Dehal, Thomas Rohan, Howard Strickler, Jessica Petrick, Marc Gunter, Xuehong Zhang, Katherine A. McGlynn, Michael Pollak, Alpa V. Patel, Susan M. Gapstur

**Affiliations:** 1 Department of Epidemiology and Population Health, Albert Einstein College of Medicine, Bronx, New York.; 2 Population Science Department, American Cancer Society (ACS), Atlanta, Georgia.; 3 Section of Nutrition and Metabolism, International Agency for Research on Cancer (IARC), Lyon, France.; 4 Division of Cancer Epidemiology and Genetics, NIH, NCI, Rockville, Maryland.; 5 Department of Clinical Science, Kaiser Permanente Bernard J Tyson School of Medicine, Panorama City, California.; 6 Slone Epidemiology Center at Boston University, Boston, Massachusetts.; 7 Channing Division of Network Medicine, Department of Medicine, Brigham and Women's Hospital and Harvard Medical School, Boston, Massachusetts.; 8 Department of Nutrition, Harvard T.H. Chan School of Public Health, Boston, Massachusetts.; 9 Depsartment of Medicine and Oncology, McGill University, Montreal, Quebec, Canada.

## Abstract

Self-reported type 2 diabetes mellitus (T2DM) is a risk factor for many cancers, suggesting its pathology relates to carcinogenesis. We conducted a case-cohort study to examine associations of hemoglobin A_1c_ (HbA_1c_) and c-peptide with cancers associated with self-reported T2DM. This study was drawn from a prospective cohort of 32,383 women and men who provided blood specimens at baseline: c-peptide and HbA_1c_ were assessed in 3,000 randomly selected participants who were cancer-free-at-baseline and an additional 2,281 participants who were cancer-free-at-baseline and subsequently diagnosed with incident colorectal, liver, pancreatic, female breast, endometrial, ovarian, bladder, or kidney cancers. Weighted Cox regression models estimated HRs and 95% confidence intervals (CI), adjusted for covariates. c-peptide was associated with higher risk of liver cancer [per SD HR: 1.80; 95% CI: 1.32–2.46]. HbA_1c_ was associated with higher risk of pancreatic cancer (per SD HR: 1.21; 95% CI: 1.05–1.40) and with some suggestion of higher risks for all-cancers-of-interest (per SD HR: 1.05; 95% CI: 0.99–1.11) and colorectal (per SD HR: 1.09; 95% CI: 0.98–1.20), ovarian (per SD HR: 1.18; 95% CI: 0.96–1.45) and bladder (per SD HR: 1.08; 95% CI: 0.96–1.21) cancers. Compared with no self-reported T2DM and HbA_1c_ < 6.5% (reference group), self-reported T2DM and HbA_1c_ < 6.5% (i.e., T2DM in good glycemic control) was not associated with risk of colorectal cancer, whereas it was associated with higher risks of all-cancers-of-interest combined (HR: 1.28; 95% CI: 1.01–1.62), especially for breast and endometrial cancers. Additional large, prospective studies are needed to further explore the roles of hyperglycemia, hyperinsulinemia, and related metabolic traits with T2DM-associated cancers to better understand the mechanisms underlying the self-reported T2DM-cancer association and to identify persons at higher cancer risk.

**Significance:**

The results from this study suggest that HbA_1c_ and c-peptide, markers of hyperglycemia and hyperinsulinemia respectively, are associated with certain cancers, though people with diabetes may be at increased risk of these cancers, perhaps other than colorectal, even when their glucose is well controlled.

## Introduction

There is ample evidence that self-reported type 2 diabetes mellitus (T2DM) is associated with increased risks of liver (1), pancreatic (2), colorectal (3, 4), female breast (5), endometrial (6), ovarian (7), bladder (8), and kidney (9) cancers. Studies that combined data from multiple, large prospective cohorts have shown relative risks of approximately 1.5 to 2.6 for cancers of the liver (1), pancreas (2), and endometrium (6) and more moderate associations for the remaining cancers, in the range of 1.2 to 1.4 (3–5, 7–9), in association with a history of self-reported T2DM. These associations have persisted after controlling for shared diabetes-and-cancer risk factors, including high body mass index (BMI), physical inactivity, smoking, and diet.

While these results are informative, studies of self-reported T2DM have important limitations toward understanding diabetes-related metabolic derangements and cancer risk. First, they do not directly address the potential carcinogenic mechanisms of hyperglycemia (e.g., via markers of glucose exposure such as hemoglobin A_1c_, HbA_1c_) or hyperinsulinemia (e.g., via markers of insulin secretion such as c-peptide). Self-reported T2DM alone is also prone to misclassification. Approximately 7.3 million of the estimated 34 million adults with diabetes in the United States are undiagnosed and would be misclassified by self-report alone; furthermore, an estimated 88 million adults in the United States have prediabetes (i.e., HbA_1c_: 5.7%–6.4%; ref. 10), a metabolic state relatively undefined in terms of its potential cancer risk. Self-reported diabetes, usually indicated by a simple “yes” response on a questionnaire, also does not allow for evaluation of good glucose control (defined as HbA_1c_ < 6.5%–7% among persons with T2DM, depending on the guideline (11, 12)) versus less-well-controlled diabetes when assessing cancer risk among persons with diabetes.

Relatively few large, prospective cohort studies have evaluated associations of biomarkers for hyperglycemia and hyperinsulinemia with risks of diabetes-associated cancers (13–26). To address this gap, we evaluated prospective associations of HbA_1c_ (an indicator of average blood glucose levels in the past 2 to 3 months) and c-peptide (an indicator of average insulin secretion in recent days/weeks) with the above-mentioned diabetes-associated cancers in a case-cohort study of 5,050 U.S. adults. For the first time in the literature, we also explored the potential influence of well-controlled diabetes, undiagnosed diabetes, and less-well-controlled diabetes on cancer risk, via variables jointly defined from self-reported T2DM and measured HbA_1c_ levels.

## Materials and Methods

### Study Participants

The Cancer Prevention Study-II (CPS‐II) Nutrition Cohort was enrolled in 1992 and 1993, in 21 states when 184,000 participants completed a mailed, self-administered questionnaire on lifestyle, behavioral, pharmacologic, medical, and sociodemographic factors. Follow‐up surveys were sent to participants beginning in 1997, and biennially thereafter, to update information and to learn of newly diagnosed cancers. Self-reported cancer diagnoses were verified by medical record abstraction or by linkage to state cancer registries. From 1998 to 2001, CPS-II Nutrition Cohort participants were invited to enroll in the CPS‐II LifeLink subcohort by providing a blood sample at a local medical facility. All LifeLink participants completed a brief questionnaire on parameters relevant to blood collection, including timing of last meal, recent medication use, diabetes status, and acute illness. Participants were not required to fast prior to blood collection. Blood samples were collected into two Ethylenediamine tetraacetic acid (EDTA) tubes and a serum separator tube. Blood samples were shipped chilled overnight to a central repository where they were fractionated and placed in liquid nitrogen freezers for long‐term storage. Further details on CPS-II Nutrition and the LifeLink subcohort are presented elsewhere (27).

For this study, we used a case-cohort study design whereby a random subcohort of 3,000 participants was selected from the 32,383 participants who provided a blood sample and did not have a prior cancer diagnosis at the time of blood draw. Next, we identified all participants who were diagnosed with one of the cancers-of-interest (28, 29) after blood draw (i.e., colorectum, *n* = 479; liver, *n* = 35; pancreas, *n* = 176; invasive female breast, *n* = 889; endometrium, *n* = 155; ovary, *n* = 93; bladder, *n* = 344; and kidney, *n* = 110) and through June 30, 2013 (the most recent data available when the study was initiated). All CPS-II LifeLink participants gave written, informed consent. CPS-II and all related substudies are approved by the Institutional Review Board at Emory University (Atlanta, GA) and all aspects of the study were conducted in accordance with the Declaration of Helsinki.

### Biomarker Measurements

Circulating biomarker concentrations were measured from red blood cell (RBC, for HbA_1c_) or serum (for c-peptide) samples from the subcohort of 3,000 participants and from the 2,281 prospectively identified cancer cases of interest (including 231 cases identified from the initial subcohort of 3,000 participants). Both biomarkers have been reliable and clinically useful when measured from nonfasting samples (30, 31), although we acknowledge c-peptide has been shown to fluctuate with timing since last meal (32). Lab personnel were blinded to case/noncase status and all plates included anonymized quality control (QC) samples.

The HbA_1c_ assay is an enzymatic measurement in which lysed whole blood or RBC samples are subjected to extensive protease digestion. The coefficient of variation (CV) for HbA_1c_ was 8.9%, with an intraclass correlation coefficient (ICC) of 74.7% for the anonymized study samples that were included on each plate. An enzymatically amplified one-step sandwich-type immunoassay measured c-peptide (Ansh labs). The CV for c-peptide was 7.7%, with an ICC of 97.5%, for the anonymized CPS-II study samples. Additional assay details are shown in Supplementary Data S1, including our QC pilot studies of: (i) HbA_1c_ reliability and validity from CPS-II long-term frozen samples, and (ii) HbA_1c_ values from frozen RBCs compared with whole blood samples from the same participants. CVs and ICCs from this latter QC experiment were 7.6% and 0.95, respectively. The mean from the frozen, fractionated RBC specimen was 4.8% whereas it was 5.2% from whole blood.

### Statistical Analyses

Weighted Cox proportional hazards regression models examined the associations of each biomarker with risk of all-cancers-of-interest combined, and with risk of the specific cancer types, with control for potential confounding variables.

HbA_1c_ was modeled continuously (per SD) and categorically (per clinical criteria for nondiabetes (referent group, HbA_1c_: <5.7%), prediabetes (HbA_1c_: 5.7%–6.4%), and T2DM (HbA_1c_ ≥ 6.5%; refs. 11, 12). c-peptide was also modeled continuously (per SD) and categorically in sex-specific tertiles.

Self-reported T2DM was recorded on the baseline questionnaire (1992 or 1993) and updated biennially beginning in 1997. Participants were asked whether they had ever been diagnosed with T2DM by a physician; beginning with the 1997 questionnaire, and for all subsequent surveys, the question also added wording to exclude persons with gestational diabetes only and the year that diabetes was diagnosed. As reported previously, self-reported T2DM was in strong agreement (90% concordant) with clinical records abstracted to confirm cancer diagnoses (33).

Covariates for multivariable models in this study were selected *a priori* and based on their potential to confound or modify the association between the biomarkers of interest and cancer risk. All covariates were collected from the questionnaires and modeled using values defined as closest time prior to or at blood draw. The covariates included in the multivariable-adjusted models were: physical activity (average hours per week of exercise: <1, 1 to <2.5, 2.5 to <4, ≥4, unknown), alcohol use (nondrinker, <1 drink per day, 1 drink per day, >1 drink per day, unknown), smoking (never, current, former), hours since last meal (<2, 2–4, 5–7, 8–11, ≥12, unknown), and hormone treatment for women (no hormone treatments, current combined estrogen and/or progestin, current estrogen only, former combined estrogen and/or progestin, former estrogen only, unknown). In addition, multivariable weighted Cox models were run with and without [calculated as weight (kg) divided by height squared (m^2^): underweight BMI: <18.5; normal BMI: 18.5 to ≤25; overweight BMI: 25 to <30; obese BMI ≥30] to show its potential confounding influence on these associations. We were unable to more finely consider the potential influence of diabetes treatments because of limited data.

Sensitivity analyses included stratifying by smoking status and, for women, by those who were current versus non-current hormone users at the time of blood draw. Additional sensitivity analyses excluded case participants diagnosed with a cancer-of-interest within 2 years after blood draw and all participants with self-reported T2DM (to avoid the potential influences of diabetes treatments and/or interventions on the biomarkers of interest with cancer risks). We also stratified bladder cancer by stage (i.e., noninvasive vs. invasive) because of previous findings for bladder cancer risk in CPS-II (34), and we stratified all cancer outcomes according to attained age (less than vs. greater than or equal to the median attained age of 78 years), by follow-up time (less than vs. greater than or equal to the median follow-up time of 9.5 years), and by age at blood draw (less than vs. greater than or equal to the median age at blood draw of 69 years). We also examined self-reported T2DM compared with no self-reported T2DM and BMI per 5 kg/m^2^, separately, with risks of the cancers-of-interest to provide broader context on the generalizability of the case-cohort participants randomly selected for this study.

To explore the potential influence of well-controlled diabetes, undiagnosed diabetes, and less-well controlled diabetes on cancer risk, we used weighted Cox proportional hazards regression models to examine cancer risk with jointly defined exposures: no self-reported T2DM and HbA_1C_ < 6.5% (reference group); self-reported T2DM with good glucose control (i.e., yes to self-reported T2DM and HbA_1c_ < 6.5%); undiagnosed diabetes (i.e., no to self-reported T2DM and HbA_1c_ ≥ 6.5%); and, less-well-controlled diabetes (i.e., yes to self-reported T2DM and HbA_1c_ ≥ 6.5%).

### Data Availability

The data underlying this article are available upon request from the corresponding author.

## Results

Descriptive characteristics for the randomly selected subcohort of 3,000 study participants with measured HbA_1c_ and 2,993 participants with measured c-peptide (seven assays failed) are shown in Table 1. HbA_1c_ and c-peptide values were higher in men than in women and for persons with versus without self-reported T2DM. HbA_1c_ and c-peptide increased directly with age and BMI. HbA_1c_ and c-peptide decreased with increasing physical activity and with moderate alcohol consumption. For both biomarkers, the lowest values were observed among current smokers compared with either former or never smokers. For the cancer outcomes identified in this study, the mean follow-up time from blood draw to diagnosis was 6.0 years (median 5.9 years, SD 3.8). For participants not diagnosed with cancer, the mean follow-up time from blood draw to end-of-study was 10.8 years (median 12.7 years, SD 3.8). Self-reported T2DM, compared with no self-reported T2DM, was associated with all-cancers-of-interest in multivariable models that included BMI [HR: 1.25; 95% confidence interval (CI): 1.04–1.49] and was also associated with higher risks of breast, endometrial, and ovarian cancers (Supplementary Table S1) whereas associations with colorectal, liver, pancreatic, and kidney cancers were suggestive of higher risks. BMI (per 5 kg/m^2^) was associated with higher risks of all-cancers-of-interest and colorectal, liver, pancreatic, breast, endometrial, and kidney cancers (Supplementary Table S2).

**TABLE 1:** Descriptive characteristics of the random subcohort of CPS-II participants with measures of c-peptide and HbA1c

	c-peptide		HbA1c	
	Total	Mean (SD)	Total	Mean (SD)
Gender				
Men	1,297	5.94 (3.16)	1,303	5.53 (1.05)
Women	1,696	5.24 (2.85)	1,697	5.39 (0.88)
Age				
<60	120	4.41 (2.41)	121	5.35 (0.87)
60–<65	544	5.40 (2.90)	544	5.35 (0.96)
65–<70	885	5.48 (3.02)	887	5.43 (1.01)
70–<75	874	5.65 (2.99)	877	5.49 (0.92)
75–<80	452	5.80 (3.12)	453	5.52 (0.91)
≥80	118	6.01 (3.27)	118	5.59 (1.01)
BMI				
<18.5	50	3.96 (2.65)	50	4.93 (0.57)
18.5–<25	1,283	4.85 (2.68)	1,286	5.29 (0.73)
25–<30	1,147	5.79 (3.11)	1,151	5.50 (1.03)
≥30	513	6.87 (3.03)	513	5.79 (1.2)
Physical activity				
<1 hour/week	709	6.04 (3.15)	709	5.60 (1.13)
1–<2.5 hours/week	535	5.92 (3.30)	537	5.43 (0.83)
2.5–<4 hours/week	765	5.41 (2.91)	768	5.48 (0.98)
≥4 hours/week	938	5.09 (2.73)	940	5.32 (0.80)
Unknown	46	4.64 (2.34)	46	5.70 (1.64)
Alcohol				
No drinks/day	1,063	5.82 (3.03)	1,066	5.58 (1.11)
<1 drink/day	1,357	5.46 (3.05)	1,360	5.42 (0.90)
1 drink/day	287	5.12 (2.67)	287	5.25 (0.69)
≥2 drinks/day	251	5.37 (2.98)	252	5.26 (0.71)
Unknown	35	4.85 (2.59)	35	5.43 (1.02)
Smoking				
Never	1,458	5.51 (3.02)	1,461	5.41 (0.87)
Former	1,436	5.61 (3.02)	1,440	5.52 (1.05)
Current	99	4.98 (2.64)	99	5.03 (0.70)
Diabetes				
No diabetes	2,662	5.46 (2.98)	2,662	5.29 (0.69)
Diabetes	331	6.19 (3.15)	338	6.71 (1.64)
Time since last ate at blood draw				
<2 hours	1,669	6.23 (3.06)	1,673	5.42 (0.92)
2–4 hours	1,075	4.90 (2.72)	1,078	5.47 (0.95)
≥5 hours	215	3.55 (2.31)	215	5.60 (1.26)
Unknown	34	4.70 (2.63)	34	5.46 (1.02)

NOTE: Data are presented as counts, arithmetic means, and SDs. Seven participants did not have c-peptide values.

Abbreviations: BMI, body mass index; HbA1c, hemoglobin A1c.

Associations between c-peptide and the cancers-of-interest in women and men combined are shown in Table 2 (sex-specific results are shown in Supplementary Table S3). Relatively high c-peptide levels were associated with higher risks of liver cancer only (HR: 4.06; 95% CI: 1.17–14.1, third vs. first tertiles), albeit with wide CIs. The remaining associations were null.

**TABLE 2:** Associations of c-peptide with risk of all cancers combined and for the specific cancers-of-interest in women and men combined in the CPS-II LifeLink cohort

	1st tertile	2nd tertile	3rd tertile	Per sex-specific SD
All sites				
Case/Total	782/1,698	715/1,625	780/1,716	./.
Model 1	1.00 (ref)	0.94 (0.81–1.08)	0.99 (0.86–1.15)	1.03 (0.96–1.09)
Model 2	1.00 (ref)	0.91 (0.79–1.05)	0.92 (0.79–1.08)	1.00 (0.93–1.06)
Colorectal				
Case/Total	172/1,149	139/1,112	168/1,167	./.
Model 1	1.00 (ref)	0.81 (0.63–1.03)	0.91 (0.71–1.18)	0.98 (0.88–1.09)
Model 2	1.00 (ref)	0.78 (0.61–1.01)	0.86 (0.66–1.12)	0.96 (0.85–1.07)
Liver				
Case/Total	4/992	11/997	20/1,036	./.
Model 1	1.00 (ref)	2.44 (0.81–7.35)	4.36 (1.46–13.0)	1.90 (1.42–2.54)
Model 2	1.00 (ref)	2.57 (0.77–8.53)	4.06 (1.17–14.1)	1.80 (1.32–2.46)
Pancreas				
Case/Total	55/1,039	59/1,041	62/1,075	./.
Model 1	1.00 (ref)	1.16 (0.79–1.71)	1.25 (0.84–1.87)	1.05 (0.90–1.23)
Model 2	1.00 (ref)	1.10 (0.74–1.62)	1.08 (0.71–1.65)	0.99 (0.84–1.16)
Breast				
Case/Total	322/848	277/807	290/834	./.
Model 1	1.00 (ref)	0.88 (0.72–1.09)	0.91 (0.73–1.13)	1.01 (0.92–1.11)
Model 2	1.00 (ref)	0.85 (0.69–1.05)	0.85 (0.68–1.06)	0.99 (0.90–1.09)
Endometrial				
Case/Total	47/391	59/397	48/398	./.
Model 1	1.00 (ref)	1.50 (0.96–2.37)	1.17 (0.71–1.93)	1.05 (0.88–1.26)
Model 2	1.00 (ref)	1.33 (0.84–2.11)	0.98 (0.58–1.66)	0.99 (0.82–1.20)
Ovarian				
Case/Total	39/471	27/446	26/469	./.
Model 1	1.00 (ref)	0.85 (0.49–1.50)	0.74 (0.40–1.38)	0.93 (0.73–1.19)
Model 2	1.00 (ref)	0.80 (0.46–1.41)	0.69 (0.38–1.28)	0.90 (0.70–1.16)
Bladder				
Case/Total	110/1,086	109/1,083	123/1,125	./.
Model 1	1.00 (ref)	0.94 (0.69–1.26)	0.99 (0.73–1.34)	1.02 (0.90–1.16)
Model 2	1.00 (ref)	0.93 (0.69–1.26)	1.00 (0.73–1.36)	1.03 (0.90–1.17)
Kidney				
Case/Total	33/1,016	34/1,022	43/1,056	./.
Model 1	1.00 (ref)	0.96 (0.58–1.58)	1.18 (0.72–1.95)	1.02 (0.84–1.25)
Model 2	1.00 (ref)	0.95 (0.57–1.57)	1.14 (0.69–1.90)	1.00 (0.82–1.23)

NOTE: Model 1: Adjusted for age, sex, smoking, physical activity, alcohol, time since last ate at blood draw, and hormone replacement therapy (HRT) (for women; men assigned same value); Model 2: Model 1 + BMI.

Associations between HbA_1c_ and the cancers-of-interest in women and men combined are shown in Table 3. HbA_1c_ ≥ 6.5%, compared with <5.7%, was statistically significantly associated with higher risk of all-cancers-of-interest combined in multivariable models that did not include BMI (HR: 1.30; 95% CI: 1.05–1.60); when BMI was added, the HR was attenuated (HR: 1.21; 95% CI: 0.98–1.50). For all-cancers-of-interest, prediabetes (HbA_1c_: 5.7%–6.4%) was associated with some suggestion of higher risk (HR: 1.11, 95% CI: 0.95–1.28) in the model that did not include BMI. In addition, HbA_1c_ ≥ 6.5%, compared with <5.7%, was statistically significantly associated with higher risk of colorectal cancer only; however, HRs for all other types of cancer, except female breast, were in the range of 1.2 to 2. Continuous HbA_1c_ (per SD) was associated with higher pancreatic cancer risk (HR: 1.21; 95% CI: 1.05–1.40).

**TABLE 3:** Associations of HbA1c with risk of all cancers combined and for the specific cancers-of-interest in women and men combined in the CPS-II LifeLink cohort

	Normal: <5.7%	Prediabetes: 5.7%–<6.5%	Diabetes: 6.5+ %	Per sex-specific SD
All sites				
Case/Total	1,660/3,716	422/916	198/417	. / .
Model 1	1.00 (ref)	1.11 (0.95–1.28)	1.30 (1.05–1.60)	1.07 (1.01–1.13)
Model 2	1.00 (ref)	1.08 (0.93–1.26)	1.21 (0.98–1.50)	1.05 (0.99–1.11)
Colorectal				
Case/Total	334/2,531	90/614	55/290	. / .
Model 1	1.00 (ref)	1.06 (0.82–1.38)	1.57 (1.13–2.18)	1.10 (1.00–1.22)
Model 2	1.00 (ref)	1.05 (0.81–1.36)	1.51 (1.08–2.10)	1.09 (0.98–1.20)
Liver				
Case/Total	21/2,244	7/540	7/248	. / .
Model 1	1.00 (ref)	1.07 (0.46–2.47)	2.24 (0.82–6.09)	1.09 (0.79–1.51)
Model 2	1.00 (ref)	0.99 (0.42–2.33)	2.02 (0.72–5.68)	1.03 (0.71–1.50)
Pancreas				
Case/Total	114/2,331	44/574	18/257	. / .
Model 1	1.00 (ref)	1.56 (1.08–2.24)	1.60 (0.94–2.70)	1.25 (1.10–1.42)
Model 2	1.00 (ref)	1.49 (1.02–2.17)	1.39 (0.82–2.38)	1.21 (1.05–1.40)
Breast				
Case/Total	698/1,935	140/400	50/154	. / .
Model 1	1.00 (ref)	1.01 (0.80–1.26)	0.95 (0.66–1.37)	0.98 (0.90–1.07)
Model 2	1.00 (ref)	0.99 (0.79–1.24)	0.88 (0.61–1.28)	0.96 (0.88–1.06)
Endometrial				
Case/Total	113/908	30/205	12/74	. / .
Model 1	1.00 (ref)	1.28 (0.82–2.00)	1.59 (0.78–3.24)	1.15 (0.98–1.36)
Model 2	1.00 (ref)	1.22 (0.78–1.93)	1.23 (0.59–2.57)	1.09 (0.91–1.31)
Ovarian				
Case/Total	66/1,058	19/238	8/92	. / .
Model 1	1.00 (ref)	1.45 (0.83–2.53)	1.80 (0.82–3.94)	1.19 (0.97–1.45)
Model 2	1.00 (ref)	1.43 (0.82–2.49)	1.78 (0.80–3.99)	1.18 (0.96–1.45)
Bladder				
Case/Total	236/2,433	72/597	36/273	. / .
Model 1	1.00 (ref)	1.06 (0.79–1.44)	1.23 (0.83–1.85)	1.07 (0.96–1.21)
Model 2	1.00 (ref)	1.07 (0.79–1.44)	1.25 (0.83–1.87)	1.08 (0.96–1.21)
Kidney				
Case/Total	78/2,298	20/553	12/250	. / .
Model 1	1.00 (ref)	0.92 (0.54–1.57)	1.23 (0.67–2.23)	1.09 (0.91–1.31)
Model 2	1.00 (ref)	0.92 (0.54–1.56)	1.17 (0.63–2.16)	1.08 (0.89–1.30)

NOTE: Model 1: Adjusted for age, sex, smoking, physical activity, alcohol, time since last ate at blood draw, and HRT; Model 2: Model 1 + BMI.

In analyses stratified by sex (Supplementary Table S4), HbA_1c_ ≥ 6.5%, compared with <5.7%, was associated with higher risk of pancreatic cancer in women in multivariable models that excluded BMI; these results were attenuated when BMI was included. In men, HbA_1c_ ≥ 6.5% was associated with risk of all-cancers-of-interest and colorectal cancer. Results from the continuous models were largely consistent with these findings.

Table 4 shows associations of the joint variable derived from self-reported T2DM and measured HbA_1c_ values. Self-reported diabetes in good metabolic control (i.e., HbA_1c_ < 6.5%), compared with the no self-reported diabetes and low HbA_1c_ (<6.5%) reference group, was associated with higher risks of all-cancers-of-interest (HR: 1.28; 95% CI: 1.01–1.62); this association was most clearly observed for breast (HR: 1.48; 95% CI: 1.02–2.15) and endometrial cancers (HR: 2.59; 95% CI: 1.26–5.32). In contrast, self-reported diabetes with good glucose control, compared with no self-reported diabetes and HbA_1c_ < 6.5%, was not associated with risk of colorectal cancer (HR: 0.95; 95% CI: 0.62–1.44) whereas both groups with high HbA_1c_, whether with undiagnosed T2DM (HR: 1.51; 95% CI: 0.91–2.52) or diagnosed T2DM with less-well-controlled diabetes (HR: 1.46; 95% CI: 0.98–2.19), had suggestive, albeit not statistically significant, increases in colorectal cancer risk. For liver and bladder cancers, undiagnosed T2DM was associated with statistically significantly higher risks, whereas undiagnosed T2DM was suggestively, but not statistically significantly, associated with all-cancers-of-interest and colorectal, pancreatic, and kidney cancers.

**TABLE 4:** Joint associations of measured HbA1c and self-reported type 2 diabetes with all cancers combined and the cancers-of-interest in the CPS-II LifeLink cohort

	HbA1c < 6.5% & no to self-reported T2DM	HbA1c < 6.5% & yes to self-reported T2DM	HbA1c ≥ 6.5+% & no to self-reported T2DM	HbA1c ≥ 6.5+% & yes to self-reported T2DM
All sites				
Case/Total	1,929/4,311	153/321	74/149	124/268
Model 1	1.00 (ref)	1.30 (1.03–1.65)	1.27 (0.92–1.77)	1.30 (1.01–1.69)
Model 2	1.00 (ref)	1.28 (1.01–1.62)	1.21 (0.87–1.69)	1.21 (0.94–1.58)
Colorectal				
Case/Total	396/2,940	28/205	20/98	35/192
Model 1	1.00 (ref)	0.96 (0.63–1.46)	1.56 (0.94–2.60)	1.53 (1.03–2.28)
Model 2	1.00 (ref)	0.95 (0.62–1.44)	1.51 (0.91–2.52)	1.46 (0.98–2.19)
Liver				
Case/Total	22/2,601	6/183	3/84	4/164
Model 1	1.00 (ref)	1.71 (0.65–4.53)	4.01 (1.05–15.4)	1.83 (0.49–6.88)
Model 2	1.00 (ref)	1.64 (0.61–4.43)	4.17 (1.13–15.5)	1.60 (0.39–6.62)
Pancreas				
Case/Total	142/2,712	16/193	7/88	11/169
Model 1	1.00 (ref)	1.49 (0.87–2.55)	1.67 (0.75–3.73)	1.38 (0.72–2.67)
Model 2	1.00 (ref)	1.46 (0.84–2.51)	1.52 (0.68–3.40)	1.18 (0.61–2.29)
Breast				
Case/Total	784/2,207	54/128	18/66	32/88
Model 1	1.00 (ref)	1.52 (1.05–2.20)	0.81 (0.46–1.43)	1.11 (0.70–1.75)
Model 2	1.00 (ref)	1.48 (1.02–2.15)	0.77 (0.43–1.35)	1.03 (0.65–1.65)
Endometrial				
Case/Total	131/1,057	12/56	4/31	8/43
Model 1	1.00 (ref)	2.80 (1.37–5.74)	1.38 (0.45–4.22)	1.78 (0.74–4.30)
Model 2	1.00 (ref)	2.59 (1.26–5.32)	1.17 (0.37–3.69)	1.33 (0.54–3.27)
Ovarian				
Case/Total	80/1,235	5/61	1/37	7/55
Model 1	1.00 (ref)	1.46 (0.57–3.74)	0.47 (0.06–3.74)	2.74 (1.15–6.50)
Model 2	1.00 (ref)	1.48 (0.58–3.83)	0.46 (0.06–3.68)	2.71 (1.10–6.68)
Bladder				
Case/Total	287/2,834	21/196	16/97	20/176
Model 1	1.00 (ref)	0.76 (0.47–1.25)	1.87 (1.02–3.42)	0.92 (0.56–1.51)
Model 2	1.00 (ref)	0.76 (0.46–1.24)	1.87 (1.02–3.43)	0.93 (0.56–1.52)
Kidney				
Case/Total	87/2,662	11/189	5/85	7/165
Model 1	1.00 (ref)	1.65 (0.84–3.23)	1.56 (0.66–3.66)	1.19 (0.55–2.59)
Model 2	1.00 (ref)	1.62 (0.83–3.15)	1.54 (0.65–3.63)	1.12 (0.51–2.46)

NOTE: Model 1: Adjusted for age, sex, smoking, physical activity, alcohol, time since last ate at blood draw, and HRT; Model 2: Model 1 + BMI.

The sensitivity and subgroup analyses were largely consistent with the main findings although we acknowledge we were underpowered for many of the stratified analyses with the rarer cancers. One exception was for bladder cancer where high HbA_1c_ was associated with risk of invasive disease (HR: 1.17; 95% CI: 1.00–1.37, per SD) and not associated with risk of noninvasive disease (HR: 1.00; 95% CI: 0.86–1.16, per SD).

## Discussion

Diabetes is a well-established major cause of macrovascular and microvascular diseases, such as heart disease, stroke, kidney failure, and blindness (10). Studies in the past 20 to 30 years further suggest increased cancer risk and mortality for people diagnosed with diabetes (28). This epidemiologic evidence relies mostly on self-reports of physician-diagnosed T2DM which has good specificity, even compared with more objective measures (33, 35); however, self-report alone generally does not identify undiagnosed T2DM, nor does it reflect the complex nature of glycemic control among people with T2DM. Given these limitations, results from this prospective study of well-characterized older adults with extensive measures of potential confounders and effect modifiers add importantly to knowledge on the associations of HbA_1c_ and c-peptide with cancer risk.

This study identified a 4-fold increased risk of liver cancer comparing the highest with the lowest tertiles of c-peptide. This finding is consistent with results from two other prospective studies that identified 3-fold increased risks of liver cancer comparing highest with lowest categories (14, 18). These results support a role for hyperinsulinemia, or its correlates, in linking self-reported T2DM to liver cancer risk. c-peptide was not associated with cancer risks other than liver cancer in this study. These null associations are consistent with the recent, albeit limited, research for female breast (13, 14), bladder (14), ovarian (14), pancreatic (14, 15), and endometrial (14, 16) cancers. Previous studies of c-peptide and colorectal cancer risk have yielded equivocal results but generally suggest an increased risk with higher c-peptide in meta-analyses and in large, prospective studies (14, 17). The relative lack of fasted blood samples in large, prospective cohort studies, such as CPS-II and others, may contribute to some difficulty in interpreting c-peptide values as a risk factor for chronic disease. We minimized this potential bias by including “time since last meal” as a covariable in our multivariable Cox proportional hazards models; however, we acknowledge that fasting samples would be superior. c-peptide values are also difficult to interpret because it conveys information on more than insulin secretion and the molecule may have pleiotropic effects, including acting as an antioxidant; in addition, low levels of c-peptide may be correlated with longer diabetes duration, pancreatic damage, and poorer glycemic control for some people with T2DM (36).

The current study shows positive associations of high HbA_1c_ with risks of all-cancers-of-interest and with colorectal cancer, although HRs for both were attenuated with the addition of BMI to the model, consistent with recent studies (17, 19). The current study also noted an association between HbA_1c_ and risk of pancreatic cancer in the continuous models as well as suggestive results for liver cancer, consistent with previous studies which also provided suggestive evidence (20–23). The lack of statistical significance with liver cancer may reflect lower statistical power. This limitation is potentially addressable in future consortium work for rare cancer types.

HbA_1c_ was not associated with risk of breast cancer in this study, despite observations of a higher risk of breast cancer with self-reported T2DM and high BMI in these same study participants. This null association between HbA_1c_ and breast cancer is consistent with most previous studies (19, 22, 24, 25), although two other studies showed positive associations (20, 26). This discordance among studies is not easily explained by an age effect, as is often observed between BMI and breast cancer risk associations stratified according to premenopausal versus postmenopausal status (37). Rather, the current findings suggest that the association between self-reported T2DM (and BMI) and breast cancer may be explained by factors other than hyperglycemia or hyperinsulinemia.

Previous research on the associations of HbA_1c_ with risks of endometrial or ovarian cancers is limited to one study of only 13 endometrial cancer cases which showed a 5-fold increased risk with high versus low HbA_1c_ (22), although the HR estimate was not adjusted for potential confounders beyond age and ethnicity. Although our results for the associations of relatively high HbA_1c_ levels with risks of ovarian or endometrial cancers were not statistically significant, the HRs were 1.78 and 1.23, respectively, after adjustment for BMI, suggestive of a potential association and warranting further study in other, large, prospective studies and pooling projects.

We did not observe statistically significant associations between HbA_1c_ and risks of either bladder or kidney cancers, although given that the HRs for high HbA_1c_ were approximately 1.2 for both cancers, we cannot rule out modest associations. We are not aware of prior publications on HbA_1c_ and risks of bladder or kidney cancers. In planned analyses, we stratified bladder cancer according to stage at diagnosis and reported a statistically significant association for invasive bladder cancer and a null association for noninvasive disease. This finding is consistent with earlier studies from CPS-II where longer T2DM duration and insulin use were associated with invasive, and not with noninvasive, bladder cancer incidence (34) and self-reported T2DM was associated with increased bladder cancer mortality (29). Our results for HbA_1c_ and kidney cancer risk were equivocal but because of relatively consistent observations of self-reported T2DM and kidney cancer risk, including suggestive findings in this subcohort (HR: 1.44; 95% CI: 0.86–2.42), future investigation of this biomarker-disease association is warranted.

A strong translational aspect of this study was the ability to assess the influence of well-controlled diabetes, less-well-controlled diabetes, and undiagnosed diabetes with cancer risks. The motivation for this joint analysis came from an earlier CPS-II publication where we noted that self-reported T2DM was associated with colorectal cancer risk in men but not in women (33), consistent with patterns reported by several other prospective studies published in that period (38–40). We interpreted the null association in women to possibly reflect better glucose control compared with that for men with T2DM, a hypothesis supported by National Health and Nutrition Examination Survey data (41). Findings from the current study support that earlier hypothesis that good glucose control among women and men with T2DM may be associated with an attenuation in colorectal cancer risk compared with T2DM with less-well-controlled HbA_1c_. These findings, if corroborated in future studies, may add colorectal cancer prevention to the list of clinical benefits from achieving glycemic targets among people with T2DM.

In contrast to the null association between good glucose control among people who self-reported T2DM and colorectal cancer risk, this study reported increased risks of all-cancers-of-interest, and especially for female breast and endometrial cancers, with this same exposure definition, an unexpected finding. In related *post hoc* analyses, the prevalence of mammography screening within 2 years of blood draw was similar across all four categories of this joint exposure (91%–94% across all categories) and women in all four exposure groups were mostly diagnosed with local staged breast or endometrial tumors. Thus, the higher risks of these cancers cannot be readily explained by a screening effect (for breast cancer) or by early detection bias (for breast or endometrial cancers) in women with T2DM and low HbA_1c_. These results underscore the increased importance for women with T2DM of being aware of signs and symptoms for endometrial and breast cancers and to undergo age-appropriate breast cancer screening.

Strengths of the current study include its relatively large sample size, prospectively collected blood specimens and cancer outcomes, repeat measures of important study variables via questionnaire (including the ability to update T2DM data), and objectively measured biomarkers. By presenting results for the eight cancer sites often reported associated with self-reported T2DM, we were able to more broadly evaluate objective biomarkers for two of the main hypotheses suspected to link T2DM to cancer risk, hyperglycemia (via HbA_1c_) and hyperinsulinemia (via c-peptide). Our QC experiments, conducted prior to launching the full study, further confirmed the validity and reliability of these assays from frozen RBCs compared with whole blood and the pilot study showed good face validity for HbA_1c_ values from RBCs stored frozen for approximately 15 years, including correlations of HbA_1c_ with BMI and T2DM in the expected directions, as well as strong CVs and ICCs from paired, frozen samples; however, we acknowledge from our QC experiments that RBC values from frozen samples were modestly lower than measures from whole blood (means of 4.8% and 5.2%, respectively) and therefore may have led to some misclassification in the overall study. Future studies should consider using fresh whole blood samples were appropriate. The hybrid variable created from self-reported T2DM and HbA_1c_ used in this study also allowed for initial exploration of the potential role of glucose control in determining cancer risk among people with T2DM, as well as the cancer risks associated with undiagnosed T2DM—both are topics for future research with other large studies or consortia projects. In addition, future pooled studies should consider a broader array of biomarkers related to metabolic health, including sex hormones and inflammatory adipocytokines.

We acknowledge the subjectivity in defining the joint T2DM-HbA_1c_ categories; for example, our referent group included people with no self-reported T2DM who had HbA_1c_ values in the prediabetes range (5.7%–6.4%). In *post hoc* analyses, excluding these participants from the referent group had no material effect on the study findings and the “no-self-reported T2DM and prediabetes” group had similar cancer rates to the referent group. Similarly, we defined HbA_1c_ values <6.5% among people with T2DM as indicative of “good diabetes control” whereas we acknowledge the most recent guidelines from the American Diabetes Association recommend a more relaxed target of 7% (42). Given the sample size limitations for case participants between an HbA_1c_ of 6.5% to 7%, we opted not to attempt another sensitivity analysis and to current findings according to our original study protocol using the cut-off point of 6.5%. This study was largely limited to older, non-Hispanic White women and men with moderate or higher education levels and these results may not be generalizable to other populations.

Perhaps, the most significant limitation of this study was the availability of samples from only one nonfasted blood draw. Although serial blood draws from prospective studies in sufficient numbers are rare, and with adequately long follow-up periods to identify cancer occurrences, we acknowledge that serial fasted blood draws for most biomarkers would be superior. Cases and noncases in this study had similar distributions for timing since last meal and blood draw, with 55.0% and 55.7% of noncases and cases, respectively, reporting eating anything within 2 hours of blood draw and only 4.1% and 3.8% of cases and noncases, respectively reporting not eating or drinking within 8 hours of blood draw. An additional limitation in this study was the relatively low sample sizes for the rarer cancers, including liver and ovarian cancers.

In conclusion, this study found little support for a link between c-peptide, a marker of endogenous insulin release, and most cancers-of-interest with the notable exception of a strong positive association with liver cancer risk. HbA_1c_, a marker of average circulating glucose over the last 2–3 months, was associated with all-cancers-of-interest and with colorectal and pancreatic cancers, specifically, in support of hyperglycemia as a mechanism linking T2DM to these cancers. Our finding that well-controlled T2DM was not associated with risk of colorectal cancer supports an earlier hypothesis (33) that good glycemic control among people with T2DM may lessen their risk of this disease relative to people with T2DM with less-well-controlled glycemia. In contrast, our finding that well-controlled T2DM was associated with higher risks of all-cancers-of-interest, largely driven by higher risks of breast and endometrial cancers, was unexpected and highlights the importance of ensuring that, regardless of glucose control, all women with diabetes receive appropriate breast cancer screening and that all men and women with diabetes are appropriately followed up for potential symptoms of diabetes-related cancers.

## Supplementary Material

Supplementary Tables S1-S4 Supplemental Table 1 - Association of self-reported T2DM at blood draw with risk of all-cancers combined and separately in CPS-II LifeLink participants; Supplemental Table 2 - Association BMI per 5 kg/m2 at blood draw with risk of all-cancers combined and separately in CPS-II LifeLink participants. Excludes those with BMI <18.5 kg/m2; Supplementary Table 3 - Associations of c-peptide with risk of all cancers combined and for the specific cancers of interest by sex in the CPS-II LifeLink cohort; Supplementary Table 4 - Associations of HbA1c with risk of all cancers combined and for the specific cancers of interest by sex in the CPS-II LifeLink cohort.

Appendix 1 lab methods and qc

## Disclaimers

- The study protocol was approved by the Institutional Review Boards of Emory University, and those of participating registries as required. The authors assume full responsibility for all analyses and interpretation of results. The views expressed here are those of the authors and do not necessarily represent the American Cancer Society or the American Cancer Society—Cancer Action Network.
- Where authors are identified as personnel of the International Agency for Research on Cancer/World Health Organization, the authors alone are responsible for the views expressed in this article and they do not necessarily represent the decisions, policy or views of the International Agency for Research on Cancer/World Health Organization.
