# Supplementary material for: Prospective Associations of Hemoglobin A1c and c-peptide with Risk of Diabetes-related Cancers in the Cancer Prevention Study-II Nutrition Cohort
Source: Cancer Res Commun. 2022 Jul 14;2(7):653–62. doi: 10.1158/2767-9764.CRC-22-0082 (PMC9881454; doi:10.1158/2767-9764.CRC-22-0082)
Supplement: Supplementary Tables S1-S4 — Supplemental Table 1 - Association of self-reported T2DM at blood draw with risk of all-cancers combined and separately in CPS-II LifeLink participants; Supplemental Table 2 - Association BMI per 5 kg/m2 at blood draw with risk of all-cancers combined and separately in CPS-II LifeLink participants. Excludes those with BMI <18.5 kg/m2; Supplementary Table 3 - Associations of c-peptide with risk of all cancers combined and for the specific cancers of interest by sex in the CPS-II LifeLink cohort; Supplementary Table 4 - Associations of HbA1c with risk of all cancers combined and for the specific cancers of interest by sex in the CPS-II LifeLink cohort. [file crc-22-0082-s01.docx]

Supplemental Table 1: Association of self-reported T2DM at blood draw with risk of all-cancers combined and separately in CPS-II LifeLink participants.

|  | | | |
| --- | --- | --- | --- |
| Cancer Site | Model | No T2DM | T2DM |
| All cancers of interest | Case/Total N | 2,009 / 4,466 | 279 / 591 |
|  | MV-HR (CI) 1 | 1.00 (ref) | 1.30 (1.08-1.55) |
|  | MV-HR (CI) 2 | 1.00 (ref) | 1.25 (1.04-1.49) |
|  |  |  |  |
| Colorectal cancer | Case/Total N | 416 / 3,038 | 63 / 397 |
|  | MV-HR (CI) 1 | 1.00 (ref) | 1.19 (0.88-1.60) |
|  | MV-HR (CI) 2 | 1.00 (ref) | 1.15 (0.85-1.56) |
|  |  |  |  |
| Liver cancer | Case/Total N | 25 / 2,685 | 10 / 347 |
|  | MV-HR (CI) 1 | 1.00 (ref) | 1.68 (0.71-3.97) |
|  | MV-HR (CI) 2 | 1.00 (ref) | 1.55 (0.62-3.91) |
|  |  |  |  |
| Pancreatic cancer | Case/Total N | 150 / 2,801 | 27 / 362 |
|  | MV-HR (CI) 1 | 1.00 (ref) | 1.40 (0.90-2.18) |
|  | MV-HR (CI) 2 | 1.00 (ref) | 1.30 (0.83-2.03) |
|  |  |  |  |
| Breast cancer | Case/Total N | 805 / 2,276 | 87 / 217 |
|  | MV-HR (CI) 1 | 1.00 (ref) | 1.36 (1.01-1.83) |
|  | MV-HR (CI) 2 | 1.00 (ref) | 1.31 (0.97-1.77) |
|  |  |  |  |
| Endometrial cancer | Case/Total N | 136 / 1,089 | 20 / 99 |
|  | MV-HR (CI) 1 | 1.00 (ref) | 2.23 (1.27-3.93) |
|  | MV-HR (CI) 2 | 1.00 (ref) | 1.90 (1.06-3.39) |
|  |  |  |  |
| Ovarian cancer | Case/Total N | 81 / 1,272 | 12 / 116 |
|  | MV-HR (CI) 1 | 1.00 (ref) | 2.05 (1.08-3.91) |
|  | MV-HR (CI) 2 | 1.00 (ref) | 2.05 (1.07-3.93) |
|  |  |  |  |
| Bladder cancer | Case/Total N | 304 / 2,932 | 41 / 372 |
|  | MV-HR (CI) 1 | 1.00 (ref) | 0.81 (0.57-1.16) |
|  | MV-HR (CI) 2 | 1.00 (ref) | 0.81 (0.56-1.15) |
|  |  |  |  |
| Kidney cancer | Case/Total N | 92 / 2,747 | 19 / 355 |
|  | MV-HR (CI) 1 | 1.00 (ref) | 1.49 (0.89-2.49) |
|  | MV-HR (CI) 2 | 1.00 (ref) | 1.44 (0.86-2.42) |

Self-reported diabetes and risk of all cancers of interest and separately in the CPS-II LifeLink prospective cohort. Multivariable-adjusted (MV) hazard ratios (HR) and 95% confidence intervals (CI): MV-HR 1 adjusted for age, gender, smoking, physical activity, alcohol, and HRT for women. MV-HR 2 additionally adjusted for BMI and all variables in MV 1.

Supplemental Table 2: Association BMI per 5 kg/m^2^ at blood draw with risk of all-cancers combined and separately in CPS-II LifeLink participants. Excludes those with BMI <18.5 kg/m^2^

| Cancer Site | MV-HR (CI) |
| --- | --- |
| All cancers of interest | 1.18 (1.10-1.27) |
| Colorectal cancer | 1.16 (1.02-1.32) |
| Liver cancer | 1.98 (1.30-3.01) |
| Pancreatic cancer | 1.44 (1.21-1.71) |
| Breast cancer | 1.12 (1.02-1.23) |
| Endometrial cancer | 1.58 (1.31-1.92) |
| Ovarian cancer | 1.11 (0.83-1.48) |
| Bladder cancer | 0.96 (0.81-1.13) |
| Kidney cancer | 1.26 (1.00-1.57) |

Body mass index per 5kg/^2^ and risk of all cancers of interest and separately in the CPS-II LifeLink prospective cohort. Multivariable-adjusted (MV) hazard ratios (HR) and 95% confidence intervals (CI): adjusted for age, gender, smoking, physical activity, alcohol, and HRT for women.

Supplementary Table 3. Associations of c-peptide with risk of all cancers combined and for the specific cancers of interest by sex in the CPS-II LifeLink cohort.

|  | Women | | | |  | Men | | | |
| --- | --- | --- | --- | --- | --- | --- | --- | --- | --- |
|  | 1^st^ tertile | 2^nd^ tertile | 3^rd^ tertile | Per sex-specific SD |  | 1^st^ tertile | 2^nd^ tertile | 3^rd^ tertile | Per sex-specific SD |
| All Sites |  |  |  |  |  |  |  |  |  |
| Case/Total | 574 / 1,082 | 510 / 1,016 | 516 / 1,037 | . / . |  | 208 / 616 | 205 / 609 | 264 / 679 | . / . |
| Model 1 | 1.00 (ref) | 0.92 (0.77-1.10) | 0.91 (0.76-1.09) | 0.99 (0.92-1.07) |  | 1.00 (ref) | 0.93 (0.73-1.18) | 1.15 (0.90-1.47) | 1.09 (0.98-1.20) |
| Model 2 | 1.00 (ref) | 0.88 (0.73-1.05) | 0.83 (0.68-1.00) | 0.96 (0.88-1.04) |  | 1.00 (ref) | 0.91 (0.72-1.17) | 1.12 (0.87-1.43) | 1.07 (0.97-1.19) |
| Colorectal |  |  |  |  |  |  |  |  |  |
| Case/Total | 98 / 650 | 81 / 633 | 79 / 647 | . / . |  | 74 / 499 | 58 / 479 | 89 / 520 | . / . |
| Model 1 | 1.00 (ref) | 0.83 (0.59-1.16) | 0.80 (0.56-1.14) | 0.92 (0.79-1.07) |  | 1.00 (ref) | 0.72 (0.49-1.05) | 1.01 (0.70-1.44) | 1.04 (0.89-1.22) |
| Model 2 | 1.00 (ref) | 0.79 (0.56-1.11) | 0.73 (0.51-1.05) | 0.86 (0.75-1.04) |  | 1.00 (ref) | 0.70 (0.47-1.02) | 0.95 (0.66-1.37) | 1.02 (0.87-1.19) |
| Liver |  |  |  |  |  |  |  |  |  |
| Case/Total | -- | -- | -- | -- |  | 2 / 431 | 10 / 436 | 16 / 456 | . / . |
| Model 1 | -- | -- | -- | -- |  | 1.00 (ref) | 3.79 (0.82-17.6) | 7.12 (1.44-35.2) | 1.97 (1.41-2.76) |
| Model 2 | -- | -- | -- | -- |  | 1.00 (ref) | 5.12 (0.86-30.7) | 8.01 (1.31-48.8) | 1.84 (1.30-2.60) |
| Pancreas |  |  |  |  |  |  |  |  |  |
| Case/Total | 27 / 585 | 26 / 585 | 33 / 608 | . / . |  | 28 / 454 | 33 / 456 | 29 / 467 | . / . |
| Model 1 | 1.00 (ref) | 1.08 (0.61-1.92) | 1.30 (0.72-2.32) | 1.08 (0.85-1.36) |  | 1.00 (ref) | 1.28 (0.74-2.23) | 1.14 (0.64-2.05) | 1.02 (0.83-1.24) |
| Model 2 | 1.00 (ref) | 0.99 (0.54-1.80) | 1.01 (0.53-1.90) | 0.96 (0.74-1.25) |  | 1.00 (ref) | 1.27 (0.73-2.20) | 1.12 (0.61-2.06) | 1.01 (0.81-1.25) |
| Breast |  |  |  |  |  |  |  |  |  |
| Case/Total | 322 / 848 | 277 / 807 | 290 / 834 | . / . |  | -- | -- | -- | -- |
| Model 1 | 1.00 (ref) | 0.88 (0.72-1.09) | 0.91 (0.73-1.13) | 1.01 (0.92-1.11) |  | -- | -- | -- | -- |
| Model 2 | 1.00 (ref) | 0.85 (0.69-1.05) | 0.85 (0.68-1.06) | 0.99 (0.90-1.09) |  | -- | -- | -- | -- |
| Endometrial |  |  |  |  |  |  |  |  |  |
| Case/Total | 47 / 391 | 59 / 397 | 48 / 398 | . / . |  | -- | -- | -- | -- |
| Model 1 | 1.00 (ref) | 1.50 (0.97-2.37) | 1.17 (0.71-1.93) | 1.05 (0.88-1.26) |  | -- | -- | -- | -- |
| Model 2 | 1.00 (ref) | 1.33 (0.84-2.11) | 0.98 (0.58-1.66) | 0.99 (0.82-1.20) |  | -- | -- | -- | -- |
| Ovarian |  |  |  |  |  |  |  |  |  |
| Case/Total | 39 / 471 | 27 / 446 | 26 / 469 | . / . |  | -- | -- | -- | -- |
| Model 1 | 1.00 (ref) | 0.85 (0.49-1.50) | 0.74 (0.40-1.38) | 0.93 (0.73-1.19) |  | -- | -- | -- | -- |
| Model 2 | 1.00 (ref) | 0.80 (0.46-1.41) | 0.69 (0.38-1.28) | 0.90 (0.70-1.16) |  | -- | -- | -- | -- |
| Bladder |  |  |  |  |  |  |  |  |  |
| Case/Total | 25 / 583 | 27 / 582 | 20 / 593 | . / . |  | 85 / 503 | 82 / 501 | 103 / 532 | . / . |
| Model 1 | 1.00 (ref) | 1.01 (0.55-1.85) | 0.68 (0.34-1.35) | 0.86 (0.66-1.18) |  | 1.00 (ref) | 0.90 (0.64-1.27) | 1.06 (0.75-1.50) | 1.06 (0.92-1.22) |
| Model 2 | 1.00 (ref) | 1.04 (0.56-1.92) | 0.74 (0.36-1.53) | 0.92 (0.68-1.26) |  | 1.00 (ref) | 0.90 (0.64-1.27) | 1.07 (0.75-1.53) | 1.06 (0.92-1.23) |
| Kidney |  |  |  |  |  |  |  |  |  |
| Case/Total | 14 / 571 | 12 / 573 | 16 / 592 | . / . |  | 19 / 445 | 22 / 449 | 27 / 464 | . / . |
| Model 1 | 1.00 (ref) | 0.72 (0.30-1.70) | 0.96 (0.40-2.29) | 0.89 (0.62-1.30) |  | 1.00 (ref) | 1.08 (0.58-2.01) | 1.33 (0.73-2.44) | 1.12 (0.89-1.42) |
| Model 2 | 1.00 (ref) | 0.64 (0.27-1.54) | 0.81 (0.34-1.95) | 0.82 (0.56-1.21) |  | 1.00 (ref) | 1.07 (0.58-1.99) | 1.31 (0.72-2.39) | 1.11 (0.88-1.41) |
|  |  |  |  |  |  |  |  |  |  |

Model 1: Adjusted for age, sex, smoking, physical activity, alcohol, time since last ate at blood draw, and HRT

Model 2: Model 1 + BMI

Supplementary Table 4. Associations of HbA1c with risk of all cancers combined and for the specific cancers of interest by sex in the CPS-II LifeLink cohort.

|  | Women | | | |  | Men | | | |
| --- | --- | --- | --- | --- | --- | --- | --- | --- | --- |
|  | Normal:  <5.7% | Pre-diabetes:  5.7-<6.5% | Diabetes:  6.5+ % | Per sex-specific SD |  | Normal: <5.7% | Pre-diabetes: 5.7-<6.5% | Diabetes:  6.5+ % | Per sex-specific SD |
| All Sites |  |  |  |  |  |  |  |  |  |
| Case/Total | 1,223 / 2,409 | 266 / 521 | 112 / 207 | . / . |  | 437 / 1,307 | 156 / 395 | 86 / 210 | . / . |
| Model 1 | 1.00 (ref) | 1.06 (0.87-1.27) | 1.18 (0.89-1.57) | 1.03 (0.96-1.10) |  | 1.00 (ref) | 1.21 (0.95-1.54) | 1.46 (1.08-1.99) | 1.17 (1.07-1.29) |
| Model 2 | 1.00 (ref) | 1.03 (0.85-1.25) | 1.07 (0.80-1.44) | 1.00 (0.93-1.07) |  | 1.00 (ref) | 1.20 (0.94-1.53) | 1.43 (1.05-1.95) | 1.17 (1.06-1.28) |
| Colorectal |  |  |  |  |  |  |  |  |  |
| Case/Total | 194 / 1,488 | 40 / 315 | 24 / 128 | . / . |  | 140 / 1,043 | 50 / 299 | 31 / 162 | . / . |
| Model 1 | 1.00 (ref) | 0.99 (0.68-1.45) | 1.59 (0.97-2.61) | 1.06 (0.93-1.21) |  | 1.00 (ref) | 1.22 (0.86-1.74) | 1.55 (1.00-2.41) | 1.15 (1.00-1.33) |
| Model 2 | 1.00 (ref) | 0.97 (0.67-1.41) | 1.50 (0.91-2.50) | 1.04 (0.91-1.20) |  | 1.00 (ref) | 1.19 (0.83-1.70) | 1.48 (0.95-2.31) | 1.14 (0.98-1.32) |
| Liver |  |  |  |  |  |  |  |  |  |
| Case/Total | -- | -- | -- | -- |  | 16 / 930 | 6 / 261 | 6 / 138 | . / . |
| Model 1 | -- | -- | -- | -- |  | 1.00 (ref) | 1.15 (0.46-2.90) | 2.68 (0.93-7.70) | 1.10 (0.71-1.69) |
| Model 2 | -- | -- | -- | -- |  | 1.00 (ref) | 1.04 (0.42-2.59) | 2.46 (0.85-7.12) | 1.03 (0.64-1.66) |
| Pancreas |  |  |  |  |  |  |  |  |  |
| Case/Total | 54 / 1,360 | 22 / 300 | 10 / 119 | . / . |  | 60 / 971 | 22 / 274 | 8 / 138 | . / . |
| Model 1 | 1.00 (ref) | 1.67 (0.98-2.82) | 2.12 (1.03-4.35) | 1.16 (0.95-1.40) |  | 1.00 (ref) | 1.37 (0.82-2.30) | 1.12 (0.51-2.46) | 1.35 (1.18-1.54) |
| Model 2 | 1.00 (ref) | 1.62 (0.95-2.76) | 1.60 (0.76-3.38) | 1.06 (0.86-1.32) |  | 1.00 (ref) | 1.37 (0.80-2.36) | 1.12 (0.51-2.47) | 1.35 (1.18-1.54) |
| Breast |  |  |  |  |  |  |  |  |  |
| Case/Total | 698 / 1,935 | 140 / 400 | 50 / 154 | . / . |  | -- | -- | -- | -- |
| Model 1 | 1.00 (ref) | 1.01 (0.80-1.26) | 0.95 (0.66-1.37) | 0.98 (0.90-1.07) |  | -- | -- | -- | -- |
| Model 2 | 1.00 (ref) | 0.99 (0.79-1.24) | 0.88 (0.61-1.28) | 0.96 (0.88-1.06) |  | -- | -- | -- | -- |
| Endometrial |  |  |  |  |  |  |  |  |  |
| Case/Total | 113 / 908 | 30 / 205 | 12 / 74 | . / . |  | -- | -- | -- | -- |
| Model 1 | 1.00 (ref) | 1.28 (0.82-2.00) | 1.59 (0.78-3.24) | 1.15 (0.98-1.36) |  | -- | -- | -- | -- |
| Model 2 | 1.00 (ref) | 1.22 (0.78-1.93) | 1.23 (0.59-2.57) | 1.09 (0.91-1.31) |  | -- | -- | -- | -- |
| Ovarian |  |  |  |  |  |  |  |  |  |
| Case/Total | 66 / 1,058 | 19 / 238 | 8 / 92 | . / . |  | -- | -- | -- | -- |
| Model 1 | 1.00 (ref) | 1.45 (0.83-2.53) | 1.80 (0.82-3.94) | 1.19 (0.97-1.45) |  | -- | -- | -- | -- |
| Model 2 | 1.00 (ref) | 1.43 (0.82-2.49) | 1.78 (0.80-3.99) | 1.18 (0.96-1.45) |  | -- | -- | -- | -- |
| Bladder |  |  |  |  |  |  |  |  |  |
| Case/Total | 60 / 1,363 | 7 / 284 | 5 / 112 | . / . |  | 176 / 1,070 | 65 / 313 | 31 / 161 | . / . |
| Model 1 | 1.00 (ref) | 0.53 (0.24-1.18) | 0.90 (0.33-2.43) | 0.92 (0.69-1.22) |  | 1.00 (ref) | 1.23 (0.88-1.73) | 1.37 (0.88-2.14) | 1.13 (0.99-1.28) |
| Model 2 | 1.00 (ref) | 0.54 (0.25-1.20) | 1.02 (0.38-2.77) | 0.95 (0.72-1.26) |  | 1.00 (ref) | 1.23 (0.88-1.73) | 1.37 (0.88-2.15) | 1.13 (0.99-1.28) |
| Kidney |  |  |  |  |  |  |  |  |  |
| Case/Total | 33 / 1,341 | 7 / 285 | 2 / 111 | . / . |  | 45 / 957 | 13 / 268 | 10 / 139 | . / . |
| Model 1 | 1.00 (ref) | 0.95 (0.41-2.22) | 0.34 (0.04-2.70) | 0.94 (0.67-1.31) |  | 1.00 (ref) | 0.89 (0.47-1.69) | 1.48 (0.75-2.94) | 1.16 (0.93-1.44) |
| Model 2 | 1.00 (ref) | 0.92 (0.40-2.13) | 0.29 (0.04-2.25) | 0.90 (0.65-1.26) |  | 1.00 (ref) | 0.89 (0.46-1.71) | 1.45 (0.72-2.92) | 1.15 (0.92-1.42) |

Model 1: Adjusted for age, sex, smoking, physical activity, alcohol, time since last ate at blood draw, and HRT

Model 2: Model 1 + BMI
