# Supplementary material for: Prospective Associations of Hemoglobin A1c and c-peptide with Risk of Diabetes-related Cancers in the Cancer Prevention Study-II Nutrition Cohort
Source: Cancer Res Commun. 2022 Jul 14;2(7):653–62. doi: 10.1158/2767-9764.CRC-22-0082 (PMC9881454; doi:10.1158/2767-9764.CRC-22-0082)
Supplement: Appendix 1 — lab methods and qc [file crc-22-0082-s02.docx]

**Appendix 1**

**Part 1: Quality control pilot testing of CPS-II Lifelink frozen red blood cell samples for hemoglobin A1c measurement.**

**Objective:** To assess quality control (QC) metrics of CPS-II Lifelink red blood cell (RBC) samples for hemoglobin A1c measurement in the laboratory of Dr. Michael Pollak at Jewish General Hospital in Montreal, QC, Canada.

**Methods:** We selected 52 participants from the Lifelink cohort who had two separate frozen RBC pelleted fraction samples. Participants were block randomly selected based on their self-reported BMI category (normal, overweight, obese) and diabetes status (type 2 diabetes mellitus: yes, no). Specifically, we selected 8 participants from each of the four normal-BMI and overweight-BMI by diabetes categories and 10 participants from each of the two obese-BMI by diabetes status categories. For each of the six BMI by diabetes categories, an equal number of men and women were selected. No other factors were considered in selecting participants.

The laboratory was sent 104 frozen samples on 31 August 2017, in four batches, blinded to BMI, diabetes, sex, and pair-matched status. Samples were thawed and assayed according to manufacturer’s instructions for HbA1c.

Laboratory data for HbA1c were returned to the Epidemiology Research Program on 24 October 2017 and assessed for intra- and inter-batch coefficients of variation (CVs, where values below 10% are considered favorable) and intraclass correlation coefficients (ICCs, a coefficient that measures the correlation between two blinded samples from the same series of participants). To assess face validity, we calculated means and standard deviations for HbA1c stratified by BMI and diabetes status categories. *A priori*, we expected to see higher HbA1c values in obese and diabetes-yes categories compared to normal-BMI and non-diabetes categories.

**Results:** All summary results are shown below. The within- and between-batch CVs were 5.4% and 2.7%, respectively, whereas the overall CV was 6%. The ICC for blinded duplicate samples from the same participants was 0.97. Mean HbA1c was higher among obese and diabetes-yes categories than among normal-BMI and non-diabetes categories. At all three levels of BMI, HbA1c was higher among participants with diabetes compared to participants who said no to diabetes. Within both diabetes categories, obese-BMI categories had higher HbA1c levels than the respective normal-BMI categories.

**Interpretation:** This pilot study indicates that this laboratory produced robust HbA1c data from Lifelink RBC specimens which have been stored, frozen, for more than 15 years. Further, the observed means and ranges of HbA1c values are within the expected physiologic ranges and correlate well with BMI and diabetes status, as anticipated.

QC Results:

Intra-batch (within batch) CV: 5.4%

Inter-batch (between batch) CV: 2.7%

Overall CV: 6.0%

ICC (intraclass): 0.967

Chart of HbA1c values by ID and batch


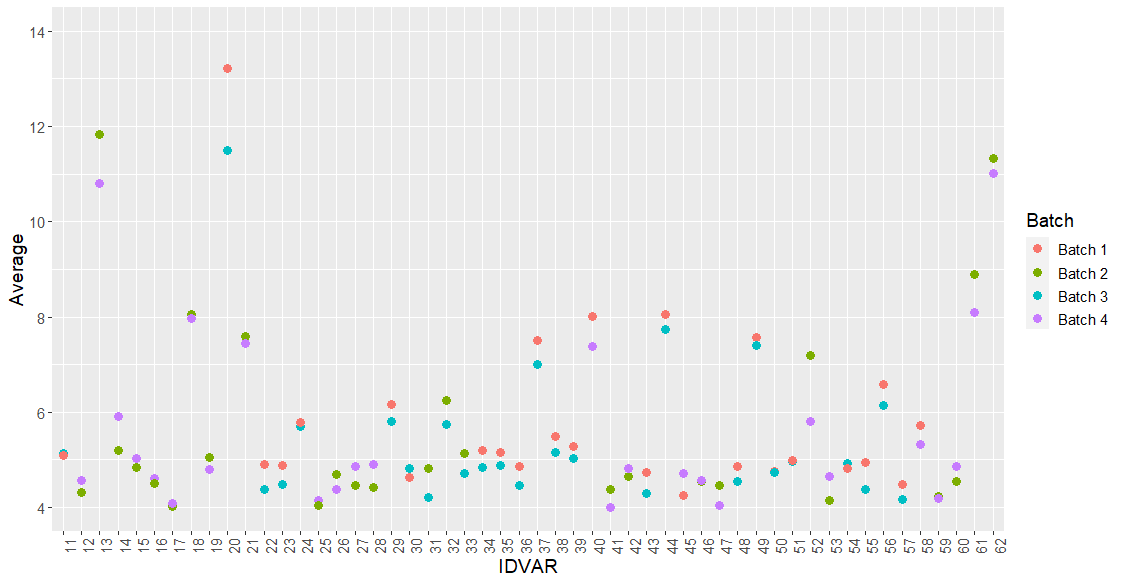


Average HbA1C values

By Weight/Diabetes Status

|  | No Diabetes | | Diabetes | |
| --- | --- | --- | --- | --- |
|  | Mean (SD) | Min, Max | Mean (SD) | Min, Max |
| Normal BMI | 4.6 (0.34) | 4.0, 5.1 | 5.9 (1.0) | 4.7, 8.1 |
| Overweight | 4.6 (0.37) | 4.0, 5.2 | 6.7 (2.6) | 4.3, 13.2 |
| Obese | 5.0 (1.0) | 4.0, 8.1 | 7.1 (2.5) | 4.4, 11.8 |

By BMI

|  | Mean (SD) | Min, Max |
| --- | --- | --- |
| Normal BMI | 5.2 (1.0) | 4.0, 8.1 |
| Overweight | 5.6 (2.1) | 4.0, 13.2 |
| Obese | 6.0 (2.2) | 4.0, 11.8 |

By Diabetes

|  | Mean (SD) | Min, Max |
| --- | --- | --- |
| No Diabetes | 4.7 (0.7) | 4.0, 8.1 |
| Diabetes | 6.6 (2.2) | 4.3, 13.2 |

**Part 2: Quality control pilot testing of whole blood versus pelleted red blood cell fractions for hemoglobin A1c measurement.**

**Objective:** To assess quality control (QC) metrics on the effect of red blood cell source on HbA1c measurement, we compared HbA1c values from the same 24 participants when HbA1c was measured in whole blood versus fractioned, red blood cell pellets. All HbA1c data were measured in the laboratory of Dr. Michael Pollak at Jewish General Hospital in Montreal, QC, Canada.

**Methods:** We identified 24 laboratory volunteers who had blood drawn and stored as two sample types: (1) whole blood and (2) the RBC fraction. HbA1c was measured from both sample types according to the assay kit manufacturer’s directions. Participants were not selected on any factors; no other information on blood donors was collected.

The blood samples were collected, and all laboratory assays were conducted in July/August 2017.

Laboratory data for HbA1c were returned to the American Cancer Society in October 2017 and assessed for overall coefficients of variation (CVs, where values below 10% are considered favorable) and intraclass correlation coefficients (ICCs, a coefficient that measures the agreement between two blinded samples from the same series of participants).

**Results:** All summary results are shown below. The overall CV was 7.66% and the ICC was 0.95 when all values were considered. When one outlier with a high HbA1c was excluded, the CV increased to 7.85% and the ICC decreased to 0.82. Mean HbA1C values from fractioned RBCs were similar to values from whole blood (mean: 4.8% versus 5.2%, respectively).

**Interpretation:** This pilot study indicates a good degree of comparability between HbA1c values from whole blood compared to pelleted fractions of RBC.

QC Results: (all one batch)

|  | All Values | Excluding Values >8% |
| --- | --- | --- |
| Overall CV | 7.66% | 7.85% |
| Pearson correlation | 0.951 | 0.822 |

Graph of HbA1C comparing Red Blood Cells vs. Whole Blood (provided by Pollak-Lab) with all values

Graph of HbA1C comparing Red Blood Cells vs. Whole Blood (provided by Pollak-Lab) excluding HbA1C over 8%

Values of HbA1C measured in Red Blood Cells vs. Whole Blood, by ID with all values


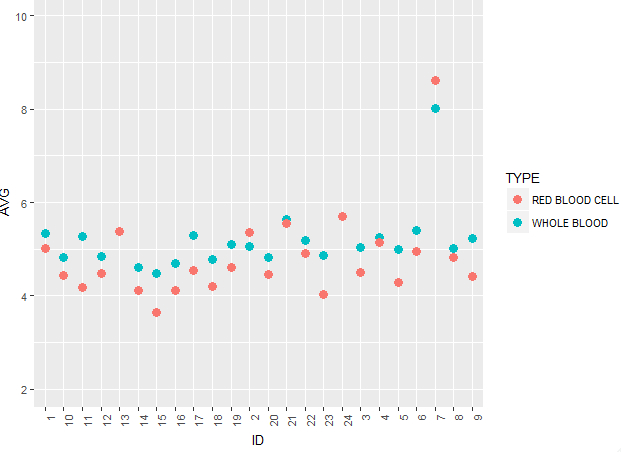


Average HbA1C in Red Blood Cells: **4.8%**, Average HbA1C in Whole Blood: **5.2%**

Values of HbA1C measured in Red Blood Cells vs. Whole Blood, by ID excluding HbA1c over 8%


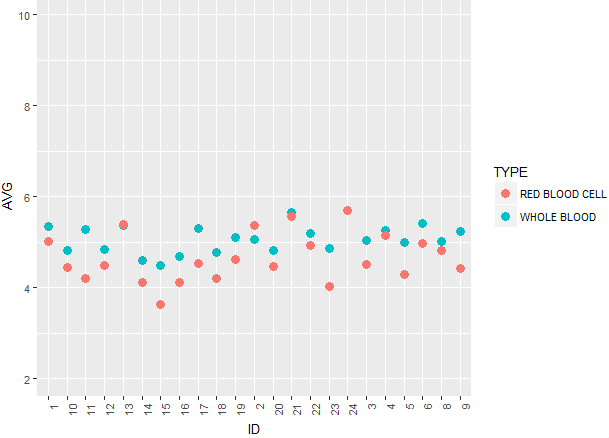


Average HbA1C in Red Blood Cells: **4.6%**, Average HbA1C in Whole Blood: **5.1%**

**Part 3: Detailed laboratory assay descriptions**

**HbA1c assay**

The HbA1c assay is an enzymatic measurement in which lysed whole blood or RBC samples are subjected to extensive protease digestion. This process releases amino acids including glycated valines from the hemoglobin beta chains. Glycated valines then serve as substrates for specific fructosyl valine oxidase (FVO) enzyme. The FVO specifically cleaves N-terminal valines and produces hydrogen peroxide. This, in turn, is measured using a horseradish peroxidase (POD) catalyzed reaction and a suitable chromagen. No separate measurement for total Hemoglobin (Hb) is needed in this direct enzymatic HbA1c assay. The required sample volume (duplicate measurement) is 20 uL of human whole blood. QC: Lyophilized human whole blood. Lab QC: Human whole blood.

**C-peptide assay**

C-Peptide of insulin ELISA is an enzymatically amplified "one-step" sandwich type immunoassay. In the assay, standards, controls and unknown serum samples are incubated with anti-c-peptide of insulin antibody in microtitration wells which have been coated with another anti-c-peptide of insulin antibody. After incubation and washing, the wells are incubated with the substrate tetramethylbenzidine (TMB). An acidic stopping solution is then added and the degree of enzymatic turnover of the substrate is determined by dual wavelength absorbance measurement at 450 and 620 nm. The required sample volume (duplicate measurement) is 40 uL of human serum or plasma. Quality control: lyophilized recombinant human c-peptide at low and high c-peptide of insulin concentrations in a protein based buffer containing Pro-Clean 400.Lab QC: Human serum or human plasma.
